# Silencing of GhSINAT5 Reduces Drought Resistance and Salt Tolerance in Cotton

**DOI:** 10.3390/genes15081063

**Published:** 2024-08-12

**Authors:** Yi Wang, Jiacong Zeng, Yuehua Yu, Zhiyong Ni

**Affiliations:** 1 Key Laboratory of Ecological Adaptation and Evolution of Extreme Environment in Xinjiang, College of Life Sciences, Xinjiang Agricultural University, Urumqi 830052, China; wangyi604987664@126.com (Y.W.); zjc2855@163.com (J.Z.); 2 College of Agronomy, Xinjiang Agricultural University, Urumqi 830052, China

**Keywords:** ubiquitin ligase (E3), SINA, VIGS, upland cotton, drought stress

## Abstract

The SEVEN IN ABSENTIA (SINA) E3 ubiquitin ligase is widely involved in drought and salt stress in plants. However, the biological function of the SINA proteins in cotton is still unknown. This study aimed to reveal the function of *GhSINAT5* through biochemical, genetic and molecular approaches. *GhSINAT5* is expressed in several tissues of cotton plants, including roots, stems, leaves and cotyledons, and its expression levels are significantly affected by polyethylene glycol, abscisic acid and sodium chloride. When *GhSINAT5* was silenced in cotton plants, drought and salinity stress occurred, and the length, area and volume of the roots significantly decreased. Under drought stress, the levels of proline, superoxide dismutase, peroxidase and catalase in the *GhSINAT5*-silenced cotton plants were significantly lower than those in the non-silenced control plants, whereas the levels of hydrogen peroxide and malondialdehyde were greater. Moreover, the expression of stress-related genes in silenced plants under drought stress suggested that *GhSINAT5* may play a positive role in the plant response to drought and salt stress by regulating these stress response-related genes. These findings not only deepen our understanding of the mechanisms of drought resistance in cotton but also provide potential targets for future improvements in crop stress resistance through genetic engineering.

## 1. Introduction

The ubiquitin proteasome system is the most important method of plant ubiquitination; ubiquitin binding to substrate proteins induces target degradation, thus altering protein function [1,2]. Ubiquitin proteins covalently bind with specific target proteins through a binding cascade of ubiquitin-activating enzyme (E1), ubiquitin-binding enzyme (E2) and ubiquitin ligase (E3) [2,3]. Compared to the relative structural conservation of E1 and E2 enzymes, E3 ligase has very high diversity. This diversity not only promotes the formation of diverse combination patterns among E1, E2 and E3 but also accurately regulates the specificity of the substrate ubiquitination process and thus closely controls complex physiological processes in cells [4]. To date, more than one thousand kinds of E3 ligase have been identified in the Plant kingdom. According to their structural characteristics, these enzymes can be classified into three main categories: the homologous to E6-associated carboxy terminus (HECT), U-box and really interesting new gene (RING) domain subfamilies. These domains are widely involved in the regulation of plant growth and development and the response to abiotic stress [5,6].

The SINA E3 ubiquitin ligase is a class of RING-type E3 ubiquitin ligases, and the N-terminal domain contains a key RING domain that specifically binds to the E2 ubiquitin-binding enzyme. The C-terminus of the protein contains the typical SINA domain, a region that not only facilitates dimerization of the SINA protein itself but is also responsible for the specific recognition and binding of the substrate protein to be degraded [7]. The earliest report of a SINA E3 ubiquitin ligase in *Drosophila* revealed that it is involved in the differentiation of R7 photocells in the *Drosophila* eye [8]. The first SINA E3 found in plants was derived from *Arabidopsis SINAT5* and is involved in lateral root development [9]. SINA genes have been found in *Arabidopsis*, poplar, rice, maize, tomatoes and other plants, and studies have shown that they play important roles in growth and development and in response to biotic and abiotic stresses [10,11]. However, only one out of three reports concerning the SINA gene in cotton was associated with abiotic stress; *GhSH1a* is an *AtSINA5* homologue with high expression in 5 days post-anthesis (DPA) fibres, and *GhSH1a*-RNAi transgenic cotton exhibited lateral root development [12]. The expression of *GhSINA7*, *GhSINA8* and *GhSINA9* increased 24 h after *Verticillium dahliae* infestation. Additionally, studies revealed that GhSINA7, GhSINA8 and GhSINA9, which have E3 ubiquitin ligase activity, are localised to the nucleus and can interact with each other; the overexpression of these genes in *Arabidopsis* increased the tolerance to *V. dahliae*, whereas their knockdown decreased the pathogen resistance in cotton [13]. Under drought stress, *GhSINA12* was overexpressed in *Arabidopsis*, and the root length of the overexpression lines under mannitol stress was significantly longer than that of the control; the opposite result occurred with virus-induced gene silencing (VIGS) of *GhSINA12*.

Cotton is not only a strategic material but also the second-largest crop after food and is a commodity that involves two major industries: agriculture and the textile industry. However, soil drought and salt stress have been exacerbated by intensified global climate change in recent years, impairing cotton growth and development [14]. The study of the gene function of cotton can help to reveal the molecular mechanism of drought resistance in cotton and cultivate drought-resistant cotton through molecular breeding. Our previous studies showed that *GhMYB4* is involved in the development of root hairs in *Arabidopsis*, overexpression of *GhMYB4* in cotton increased drought tolerance and salt tolerance in cotton, and yeast two-hybrid experiments revealed that GhSINAT5 interacts with GhMYB4 [15,16]. Therefore, we hypothesised that *GhSINAT5* also plays an important role in drought and salt tolerance in cotton. In this study, the expression of *GhSINAT5* was induced by drought, abscisic acid (ABA) and sodium chloride (NaCl) stress, and silencing *GhSINAT5* reduced cotton resistance by increasing the degree of cell membrane damage, reducing antioxidant enzyme activity and the expression levels of the drought-related genes *GhNCED3*, *GhRD22* and *GhRD26*.

## 2. Materials and Methods

### 2.1. Plant Materials

Y1169, a line that is widely planted in cotton-producing areas of Xinjiang, was used for quantitative real-time polymerase chain reaction (qPCR) analysis and as a transient conversion receptor for VIGS. Cotton seeds with a full shape were selected, soaked in 30% hydrogen peroxide solution (H_2_O_2_) for 3–4 h (h), cleaned with distilled water 3–5 times, placed in germination boxes with filter paper and incubated for 2 days (d) at 28 °C. White cotton seeds were selected and planted in black soil containing 50% vermiculite (black soil:vermiculite, 1:1) and covered with film; the plants were allowed to germinate for one week. After the seedlings were completely germinated, the film was removed, and the seedlings were cultured at 28 °C with a 16 h light/8 h dark cycle until they reached the “2 leaves and 1 heart” stage (Light Incubator, Ningbo Jiangnan Instrument Factory, Ningbo, China). The roots, stems, leaves and cotyledons were collected. The samples were flash-frozen with liquid nitrogen and stored at −80 °C.

For hydroponic cotton: The 3-leaf cotton plants were transferred to hydroponics and allowed to recover for 2 d before being immersed in 15% PEG 6000, 250 mM NaCl or 100 µM ABA for 1 week [15]. Samples of cotton leaves were collected at 0 h, 2 h, 4 h, 6 h, 12 h and 24 h.

### 2.2. Extraction of Total Cotton RNA and Synthesis of the First Strand of cDNA

The method for extracting total cotton RNA was performed with a RNAplant Plus Kit (Tiangen Biochemical Technology (Beijing) Co., Ltd., Beijing, China). The first strand of cDNA was synthesised using the FastKing cDNA First-strand Synthesis Kit (Beijing, Tiangen Biochemical Technology (Beijing) Co., Ltd.), which removes genomic DNA contamination. The reverse-transcribed cDNA was used for vector construction and real-time fluorescence quantitative reaction.

### 2.3. Quantitative Real-Time Polymerase Chain Reaction

qPCR primers were designed based on the *GhSINAT5* gene sequences obtained by sequencing, and the cotton *GhUBQ7* gene was used as an internal reference [14]. Amplification was performed using an ABI 7500 fast real-time PCR instrument in a reaction volume of 20 μL. The qPCR amplification system and amplification procedures were performed according to the instructions of the fluorescence quantification kit. The cycle threshold (Ct) of each sample was tested, and the experimental data were analysed with the 2^−∆∆Ct^ method; each experiment was performed with three biological replicates. The transcript levels of the *GhSINAT5* genes in different cotton tissues were analysed, and the roots were used as control samples. The primer sequences are listed in Supplementary Table S1.

### 2.4. Gene Cloning and Bioinformatics Analysis

Cloning primers were designed based on the *GhSINAT5* gene sequence published in the cotton gene database. The first strand of fibre cDNA (1 DPA) from Y1169 was used as a template to generate the *GhSINAT5* gene. The amplified *GhSINAT5* gene was connected to the pMD 18-T vector to obtain the GhSINAT5 open reading frame (ORF) sequence.

The molecular weight (MW), isoelectric point (pI) and homology of the GhSINAT5 protein were predicted using DNAMAN7 software. Multiple sequence alignment was performed via ClustalX 1.83 software.

The key drought stress-related genes, including the ABA biosynthesis gene 9-cis-epoxycarotenoid dioxygenase 3 (*NCED3*), drought-induced *RD22* gene and drought-induced *RD26* gene, encoding the NAC transcription factor, were obtained from the cotton database (https://cottonfgd.org, accessed on 26 August 2023.).

### 2.5. VIGS and Gene Expression Level Detection

DNA from upland cotton Y1169 leaves as a template was amplified by PCR using primers at the *EcoR*I and *BamH*I restriction sites (Supplementary Table S1), and the amplified product and empty vector TRV::00 were double-digested, linked and sequenced to obtain TRV::00-GhSINAT5. The TRV::00-GhSINAT5 plasmid was extracted and transferred into Agrobacterium GV3101 via the freeze‒thaw method. The TRV::00-GhSINAT5 recombinant plasmid, TRV::GhCLA1 (positive control) and TRV::00-pTRV2 (negative control) were mixed at a ratio of 1:1 with a resuspension of TRV::00-pTRV1 and injected into the cotyledons at the time of two leaves and one heart, respectively [17]. The expression of GhSINAT5 in TRV::00-GhSINAT5 plants was measured by qPCR 15 d after injection. Three replicates of this experiment were performed, with 20 cotton plants in each replicate.

### 2.6. Cotton Stress Treatment

When the cotton reached the 3–4-leaf stage, the TRV::00-GhSINAT5-silenced plants and TRV::00 negative control plants were subjected to 15% polyethylene glycol (PEG) stress and natural drought stress [14]. The phenotypes were recorded 36 h after PEG stress treatment. Phenotypic differences after approximately 10 d of drought stress treatment were recorded, and the survival rate after rehydration for 3 d was calculated.

### 2.7. Determination of Physiological and Biochemical Indicators

The second or third true leaves of TRV::00-GhSINAT5-silenced plants and TRV::00 negative control plants before and 10 d after drought stress were selected, and physiological and biochemical index detection kits from Nanjing Jiancheng Technology Co., Ltd. (Nanjing, China), were used to measure the contents of proline (Pro), malondialdehyde (MDA) and H_2_O_2_ and the activities of the antioxidant enzymes (catalase [CAT], superoxide dismutase [SOD] and peroxidase [POD]).

### 2.8. Statistical Data

GraphPad Prism 9.0 (GraphPad Software, San Diego, CA, USA) was used to perform *t*-tests, chi-square tests, ANOVA and graphs.

## 3. Results

### 3.1. Cloning and Sequence Analysis of GhSINAT5

**Figure 1:**
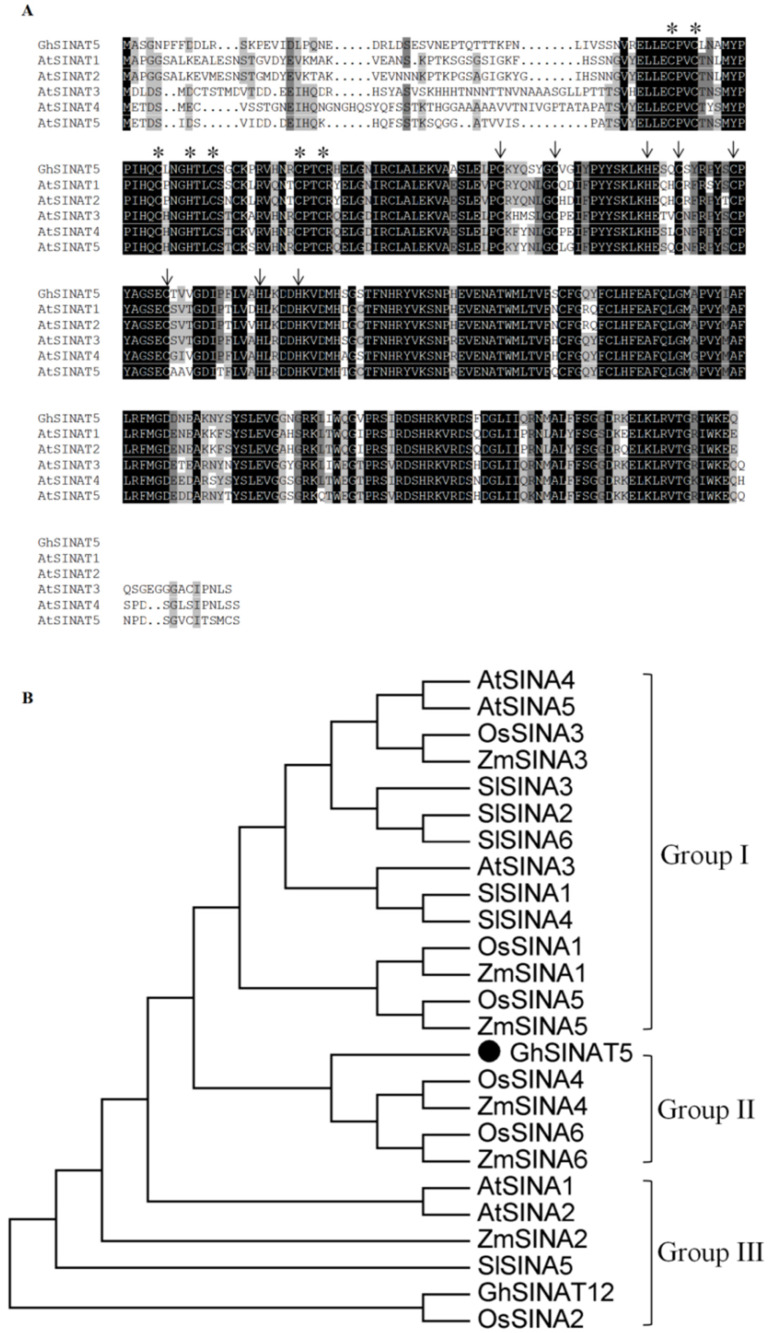
The GhSINAT5 phylogenetic tree and analysis of the conserved domains. (**A**) Multiple sequence alignment of GhSINAT5 and *Arabidopsis*. (**B**) Phylogenetic tree analysis of the SINA ubiquitin ligases in GhSINAT5, Arabidopsis, maize, rice and tomatoes. Asterisks and arrows indicate conserved amino acids in the RING and B-box2 conserved domains.

Sequence analysis of the GhSINAT5 cDNA (1640 bp with a 915 bp ORF) revealed the physiochemical properties and structural and functional domains via bioinformatics. The cotton *GhSINAT5* gene encodes a protein of 304 amino acids with a molecular weight of approximately 34.62 kDa and a theoretical isoelectric point of 7.88. Domain prediction revealed that the GhSINAT5 protein contains a RING-HC-type zinc finger domain at sites 54–92 and a SINA domain at sites 98–297 (Figure 1A). Phylogenetic tree analysis revealed that GhSINAT5 is in Group II; has high similarity with the ZmSINA4, ZmSINA6, OsSINA4 and OsSINA6 proteins; is in the same clade and recently diverged (Figure 1B).

### 3.2. GhSINAT5 Expression Pattern Analysis

**Figure 2:**
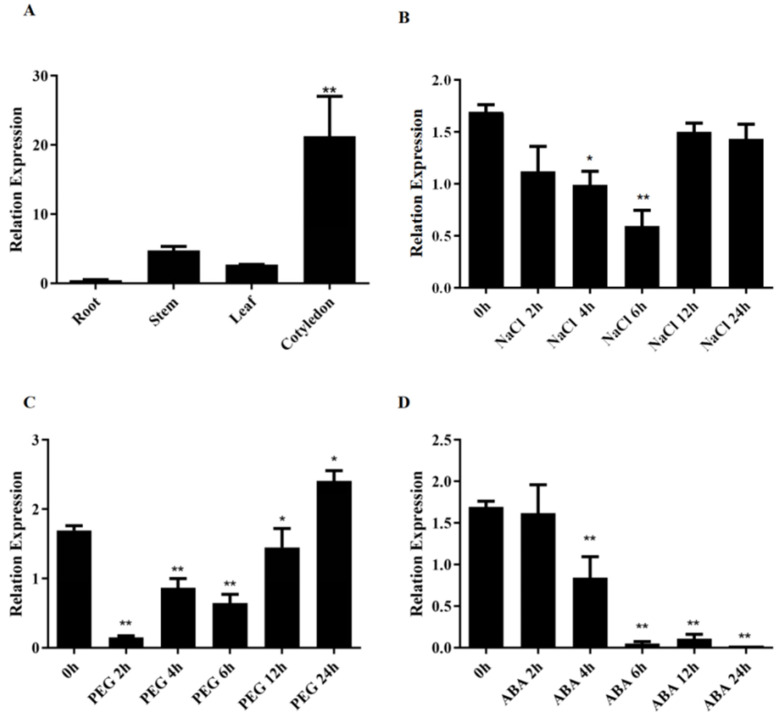
Analysis of the GhSINAT5 transcript levels. (**A**) Analysis of the transcript levels in different tissues, with roots as the control. (**B**) Expression patterns at different periods after 250 mM NaCl treatment. (**C**) Expression patterns at different periods after 15% PEG treatment. (**D**) Expression patterns at different periods after 100 μM ABA treatment. Vertical bars indicate ±SDs, and significant differences from the control are indicated as * *p* < 0.05 and ** *p* < 0.01.

Roots, stems, leaves and cotyledons are collected when cotton reaches the “2 leaves and 1 heart” stage. The expression of the *GhSINAT5* gene in different tissues was investigated using qPCR, which showed that the highest expression of *GhSINAT5* occurred in cotyledons (22 times), followed by stem (5 times) and leaf (3 times) tissues, and the lowest expression occurred in roots (1 time) (Figure 2A).

To investigate whether *GhSINAT5* responds to drought stress, cotton leaves were collected at different time points (0 h, 2 h, 4 h, 6 h, 12 h and 24 h) after 15% PEG, 250 mM NaCl and 100 μM ABA treatments when the cotton had grown to two leaves and one heart. Expression analysis using qPCR revealed that *GhSINAT5* exhibited different responses under different stresses. Under NaCl stress, the expression of the *GhSINAT5* gene started to decrease at 2 h, decreased significantly from 4 h to 6 h and reached the lowest expression at 6 h, then increased after 6 h, and there was no significant change in the expression at 12 h and 24 h compared to the first 0 h (Figure 2B). *GhSINAT5* expression significantly decreased after 2 h, 4 h, 6 h and 12 h of PEG treatment and significantly increased after 24 h (Figure 2C). Under ABA stress, *GhSINAT5* expression did not significantly change after 2 h of ABA stress, followed by a significant decrease in its expression level during the 4–24 h period (Figure 2D).

### 3.3. Silencing GhSINAT5 Reduces Drought Resistance in Cotton

**Figure 3:**
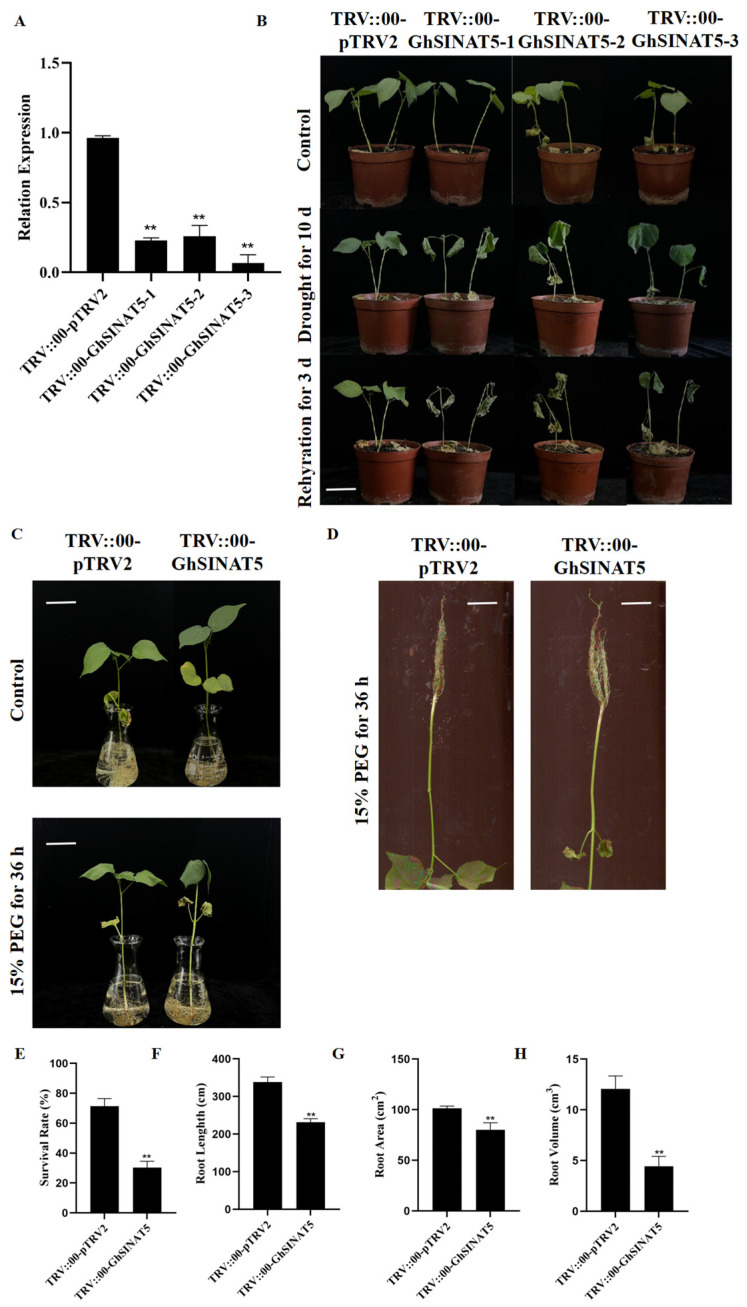
Identification of drought-resistant phenotypes of cotton with silencing of the GhSINAT5 gene. (**A**) GhSINAT5 gene expression assay. (**B**) Phenotypic analysis after 10 d of natural drought. (**C**) The 15% PEG-simulated drought phenotype. (**D**) Root length phenotype under 15% PEG drought stress. (**E**) Survival rate statistics under drought stress. (**F**) Root length under drought stress. (**G**) Root area under drought stress. (**H**) Root volume under drought stress. TRV::00-pTRV2 indicates control, TRV::00-GhSINAT5 indicates silent plants, vertical bars indicate ±SDs and significant differences from the control are indicated as ** *p* < 0.01.

The GhSINAT5 gene was silenced in cotton by VIGS. Resuspension solutions of TRV::00-GhSINAT5, the positive control TRV::GhCLA1 and the negative control TRV::00-pTRV2 were mixed with the resuspension solution of TRV::00-pTRV1 at a 1:1 ratio and then injected into the cotyledons at the two-leaf one-heart stage of cotton. The plants were cultured in the dark for 1 d and then transferred to a normal culture at room temperature (28 °C). Approximately 14 d after injection, cotton plants harbouring the marker gene TRV::GhCLA1 started to exhibit an albino phenotype in their true leaves (Figure S1). The expression of the TRV::00-GhSINAT5-silenced plants and TRV::00-pTRV2 control plants was measured using qPCR, as shown in Figure 3A. Compared to that of the control plants, the expression of TRV::00-GhSINAT5 in the cotton plants was significantly lower, suggesting that the target genes were successfully silenced.

The TRV::00-GhSINAT5 plants and TRV::00-pTRV2 control plants were phenotypically similar in the absence of any treatment. However, after 10 d of simultaneous drought stress treatment, both the TRV::00-GhSINAT5 plants and the control plants experienced varying degrees of wilting; compared to the control plants, the TRV::00-GhSINAT5 plants presented more severe wilting, leaf yellowing, leaf abscission and even death (Figure 3B). The survival statistics of the TRV::00-GhSINAT5 gene-silenced plants and TRV::00-pTRV2 control plants after 3 d of rehydration revealed that the survival rate of the TRV::00-pTRV2 negative control plants was 71.45% and that of the TRV::00-GhSINAT5 gene-silenced plants was 30.3%, which was significantly lower (Figure 3E). After 36 h of 15% PEG stress treatment, the control plants were well grown compared to the TRV::00-GhSINAT5 plants, which exhibited more severe wilting (Figure 3C). The root systems of TRV::00-pTRV2 and TRV::00-GhSINAT5 plants under 15% PEG stress were examined, and TRV::00-GhSINAT5 plants presented significantly lower root lengths, root areas and root volumes than TRV::00-pTRV2 plants (Figure 3D,F–H). The above results tentatively indicate that silencing the GhSINAT5 gene reduces drought tolerance in cotton.

### 3.4. Silencing of GhSINAT5 Increases the Extent of Membrane Injury and Decreases Antioxidant Enzyme Activity in Cotton Cells

**Figure 4:**
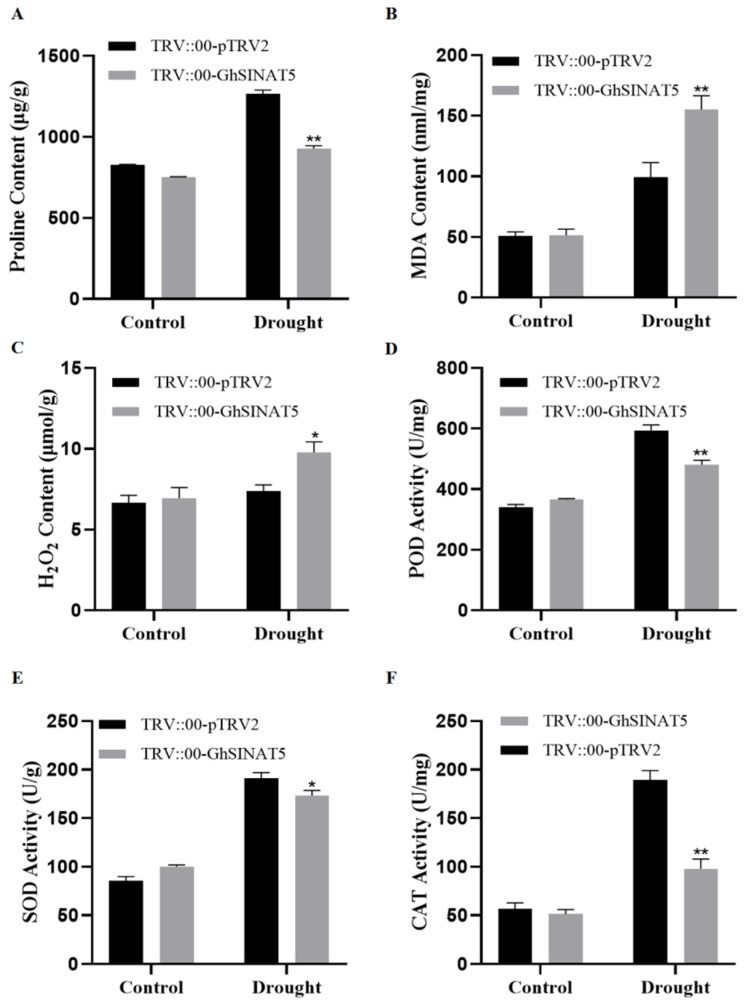
Measurement of the physiological and biochemical indices under drought stress in cotton with silencing of the GhSINAT5 gene. (**A**) Proline content. (**B**) Malondialdehyde content. (**C**) Hydrogen peroxide content. (**D**) Peroxide dismutase content. (**E**) Superoxide dismutase content. (**F**) Catalase content. TRV::00-pTRV2 indicates control, TRV::00-GhSINAT5 indicates silent plants, vertical bars indicate ±SDs and significant differences from the control are indicated as * *p* < 0.05 and ** *p* < 0.01.

To test whether silencing of the GhSINAT5 gene affects the physiological and biochemical indices of cotton, the Pro, MDA, H_2_O_2_, CAT, POD and SOD levels were determined before and after drought stress induction in the control plants and TRV::00-GhSINAT5 plants. There was no significant difference in Pro levels between the control and TRV::00-GhSINAT5 plants before stress treatment (Figure 4A). After 10 d of drought stress, the Pro levels increased in both groups, but the Pro levels of TRV::00-pTRV2 plants were greater than those of TRV::00-GhSINAT5 plants (Figure 4A). In the absence of any treatment, the MDA and H_2_O_2_ levels were similar in TRV::00-pTRV2 and TRV::00-GhSINAT5 plants (Figure 4B,C). After 10 d of drought treatment, the MDA and H_2_O_2_ levels were increased in both groups, but the levels in TRV::00-pTRV2 plants were lower than those in TRV::00-GhSINAT5 plants (Figure 4B,C).

The results of the CAT, POD and SOD activity measurements revealed that the antioxidant enzyme activities were similar between the control and TRV::00-GhSINAT5 plants before drought stress treatment (Figure 4D–F). The antioxidant enzyme activities of the TRV::00-GhSINAT5 plants and control plants increased to different degrees after 10 d of drought stress treatment. The CAT, POD and SOD activities were significantly lower in the TRV::00-GhSINAT5 plants than in the control plants (Figure 4D–F).

### 3.5. GhSINAT5 Regulates the Expression of Key Genes Involved in Drought Stress

**Figure 5:**
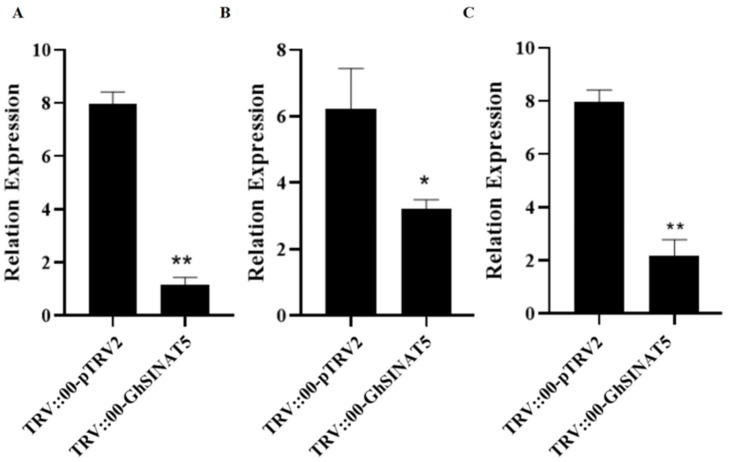
GhNCED3, GhRD22 and GhRD26 gene expression assays. (**A**) Expression of GhRD22. (**B**) Expression of GhRD26. (**C**) Expression of GhNCED3. TRV::00-pTRV2 indicates control, TRV::00-GhSINAT5 indicates silent plants, vertical bars indicate ±SDs and significant differences from the control are indicated as * *p* < 0.05 and ** *p* < 0.01.

To elucidate the molecular mechanisms of GhSINAT5 in response to drought stress, the expression levels of GhNCED3, GhRD22 and GhRD26 were analysed by qPCR in the TRV::00-GhSINAT5 plants and control plants after drought stress. The results revealed significantly lower expression of the GhNCED3, GhRD22 and GhRD26 genes in the TRV::00-GhSINAT5 plants than in the control plants (Figure 5).

### 3.6. Silencing of GhSINAT5 Reduces Salt Resistance in Cotton

**Figure 6:**
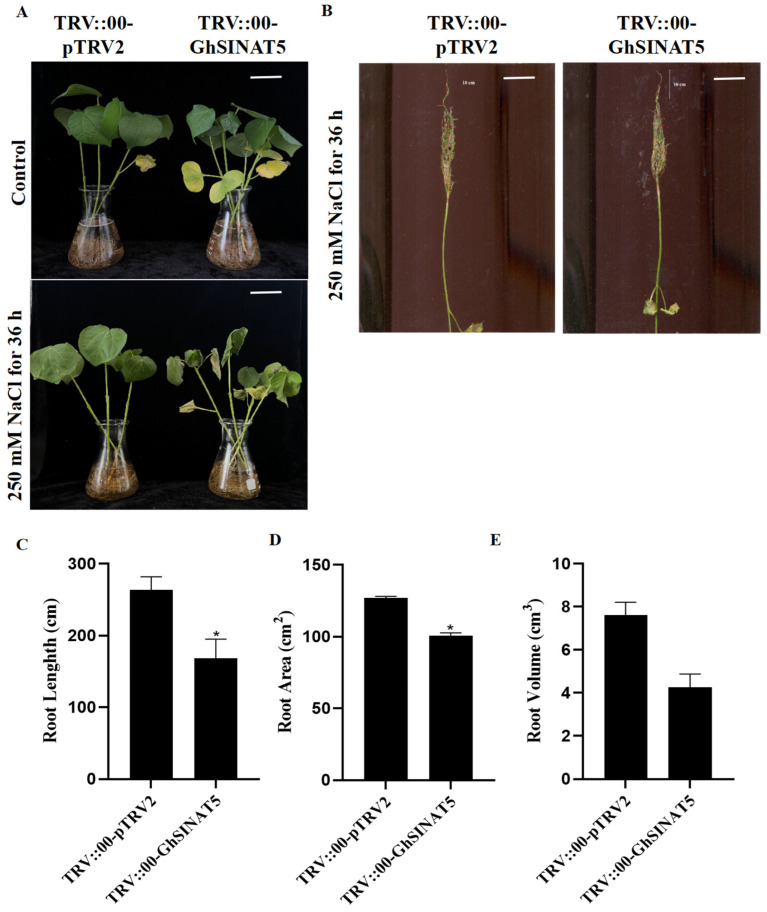
Identification of the salt stress phenotype of cotton with silenced GhSINAT5 genes. (**A**) NaCl stress phenotype at 250 mM. (**B**) NaCl stress-induced root length at 250 mM. (**C**) Root length under NaCl stress at 250 mM. (**D**) Root area under NaCl stress at 250 mM. (**E**) Root volume under NaCl stress at 250 mM. TRV::00-pTRV2 indicates control, TRV::00-GhSINAT5 indicates silent plants, vertical bars indicate ±SDs and significant differences from the control are indicated as * *p* < 0.05.

The TRV::00-GhSINAT5 plants and TRV::00-pTRV2 negative control plants were subjected to 250 mM NaCl stress. Before NaCl treatment, the TRV::00-GhSINAT5 plants and TRV::00-pTRV2 control plants were phenotypically similar; after 36 h of treatment, the TRV::00-GhSINAT5 plants presented more severe leaf yellowing, wilting and even death than the control plants (Figure 6A). The TRV::00-GhSINAT5 plants had significantly lower root lengths, root areas and root volumes than the TRV::00-pTRV2 plants (Figure 6B–E). These results indicated that silencing the GhSINAT5 gene in cotton reduced its salt tolerance.

## 4. Discussion

An increasing number of studies have shown that, in addition to regulating plant growth and development, the expression of SINA E3 ubiquitin ligase-encoding genes is also induced by drought and salt stress. For example, expression of the gene encoding the wheat SINA E3 ubiquitin ligase *TaDIS1* was induced by PEG, ABA and NaCl stress treatments [18]. *TaSINA2B* is induced by PEG, ABA and osmotic stress. In a previous study, *GhSINA7A* (i.e., *GhSINAT5*) was found to be a positive regulator of yellow wilting, and its expression was induced by drought stress [13]. Similar to the results of the previous study, our study also revealed that *GhSINAT5* expression was induced by drought stress, but the expression trend of *GhSINA7A* after 24 h of drought stress was opposite to that of *GhSINAT5*, probably due to the different cotton varieties used. In addition, *GhSINAT5* was induced by ABA and NaCl stress.

Previous studies have shown that SINA E3 can either positively or negatively regulate drought tolerance in plants [19,20,21]. OsDIS1 represses drought tolerance in rice and interacts with OsSKIPa to regulate the expression of downstream adversity-responsive genes, thereby modulating drought tolerance in rice [19,20]. However, *AtSINA2* improved plant drought resistance, whereas, under drought conditions, *AtSINA2* promoted ABA-mediated leaf stomata closure, reducing water loss and thus improving the drought resistance of transgenic plants [21]. Compared to that in the wild-type strain, heterologous overexpression of *GhSINA12* in *Arabidopsis thaliana* resulted in significantly longer roots, and the silencing of the *GhSINA12* gene through VIGS resulted in more severe wilting of the *GhSINA12*-silenced cotton plants than in the control plants after drought stress, as well as significantly lower SOD, POD and CAT activities; conversely, the silenced plants presented significantly higher MDA levels than those in the control plants. Similar to *GhSINA12*-silenced plants, the *GhSINAT5*-silenced plants wilted more severely than the control plants did under drought stress, and the length, area and volume of the roots in the *GhSINAT5*-silenced plants were significantly shorter than those in the control plants. Moreover, the SOD, POD, CAT and MDA levels of the *GhSINAT5*-silenced plants showed the same trends as those of the *GhSINA12*-silenced plants. In addition, silencing *GhSINAT5* also affected the levels of Pro and H_2_O_2_. There have been relatively few studies on the involvement of the SINA E3 ubiquitin ligases in salt stress. The overexpression of *BnaSINA17* in *Arabidopsis sina2* mutants restores resistance to salt stress and osmotic stress [22]. Our results revealed that silencing *GhSINAT5* reduced not only drought tolerance but also salt tolerance in cotton, and the silenced plants wilted more severely than the control plants under salt stress and had significantly shorter root lengths, root areas and root volumes than the control.

*GhSINAT5* responds to ABA stress, and *NCED3* is a key gene for ABA synthesis [23]. Previous studies have shown that *AtRD22* and *AtRD26* are abiotic stress response marker genes in *Arabidopsis* [24,25]. We identified terrestrial cotton homologues of the above genes, and the transcript levels of *GhNCED3*, *GhRD22* and *GhRD26* were significantly reduced in *GhSINAT5*-silenced cotton plants after drought stress.

Although the specific molecular mechanism of *GhSINAT5* in drought and salt stress has not yet been fully elucidated, the results of this study have improved our understanding of the mechanisms of plant adversity adaptation and provide an important theoretical basis and practical guidance for enhancing the drought and salt stress tolerance of plants through molecular breeding techniques and genetic engineering in the future.

## 5. Conclusions

In summary, the expression of the gene encoding GhSINAT5, a member of the SINA E3 ubiquitin ligase family, was induced by drought, ABA and NaCl stress conditions. Further studies revealed that, when *GhSINAT5* was silenced in cotton, it reduced the drought and salt tolerance of the plant by exacerbating the degree of damage to cotton cell membranes, weakening the activities of antioxidant enzymes (e.g., SOD, POD and CAT) and affecting the expression patterns of a series of stress response-related genes. These findings strongly suggest that *GhSINAT5* functions as a positive regulator in response to drought and salt stress, and it can be used as a candidate gene to improve drought and salt tolerance in plants.

## Data Availability

All data generated during this study are included within the article or its supplementary files.

## Supplementary Materials

The following supporting information can be downloaded at https://www.mdpi.com/article/10.3390/genes15081063/s1: Figure S1: The albino phenotype. Table S1: Primer sequences used in this study.
