# Supplementary material for: Silencing of GhSINAT5 Reduces Drought Resistance and Salt Tolerance in Cotton
Source: Genes (Basel). 2024 Aug 12;15(8):1063. doi: 10.3390/genes15081063 (PMC11353778; doi:10.3390/genes15081063)
Supplement: Supplementary file 1 [file genes-15-01063-s001.zip › Table S1.pdf]

Table S1 Primer sequences used in this study

**Name**	**Sequence**
GhSINAT5-qF	5'-CCTTCGCAGCTTGGTCCTAA-3'
GhSINAT5-qR	5'-AAATGGGGTAGGAGGGGTGA-3'
GhUBQ7-F	5'-GACCTACACCAAGCCCAAGAAG-3'
GhUBQ7-R	5'-TGAGCCCACACTTACCACAATAGT-3'
GhSINAT5-TRV2-F	5'-CCGGAATTC GTAATAGACAGTAGATCGGTTAC-3'
GhSINAT5-TRV2-R	5'-CGGGATCCTGAGCGTTGATCAACGATAGTTG-3'
GhRD22-F	5'-AGAGGGTGCTGACGGAACAA-3'
GhRD22-R	5'-TGCTTAGGGTTCCATGCGGA-3'
GhRD26-F	5'-GCGAGCTGATAACTCGGGGA-3'
GhRD26-R	5'-GTCGGGCACCGAAACCCATA-3'
GhNCED3-F	5'-CCTGCATGACACCCCCAGAT-3'
GhNCED3-R	5'-TCGACTTGCCGGTCCTCAAA-3'
