# Supplementary figures and images for: Silencing of GhSINAT5 Reduces Drought Resistance and Salt Tolerance in Cotton
Source: Genes (Basel). 2024 Aug 12;15(8):1063. doi: 10.3390/genes15081063 (PMC11353778; doi:10.3390/genes15081063)

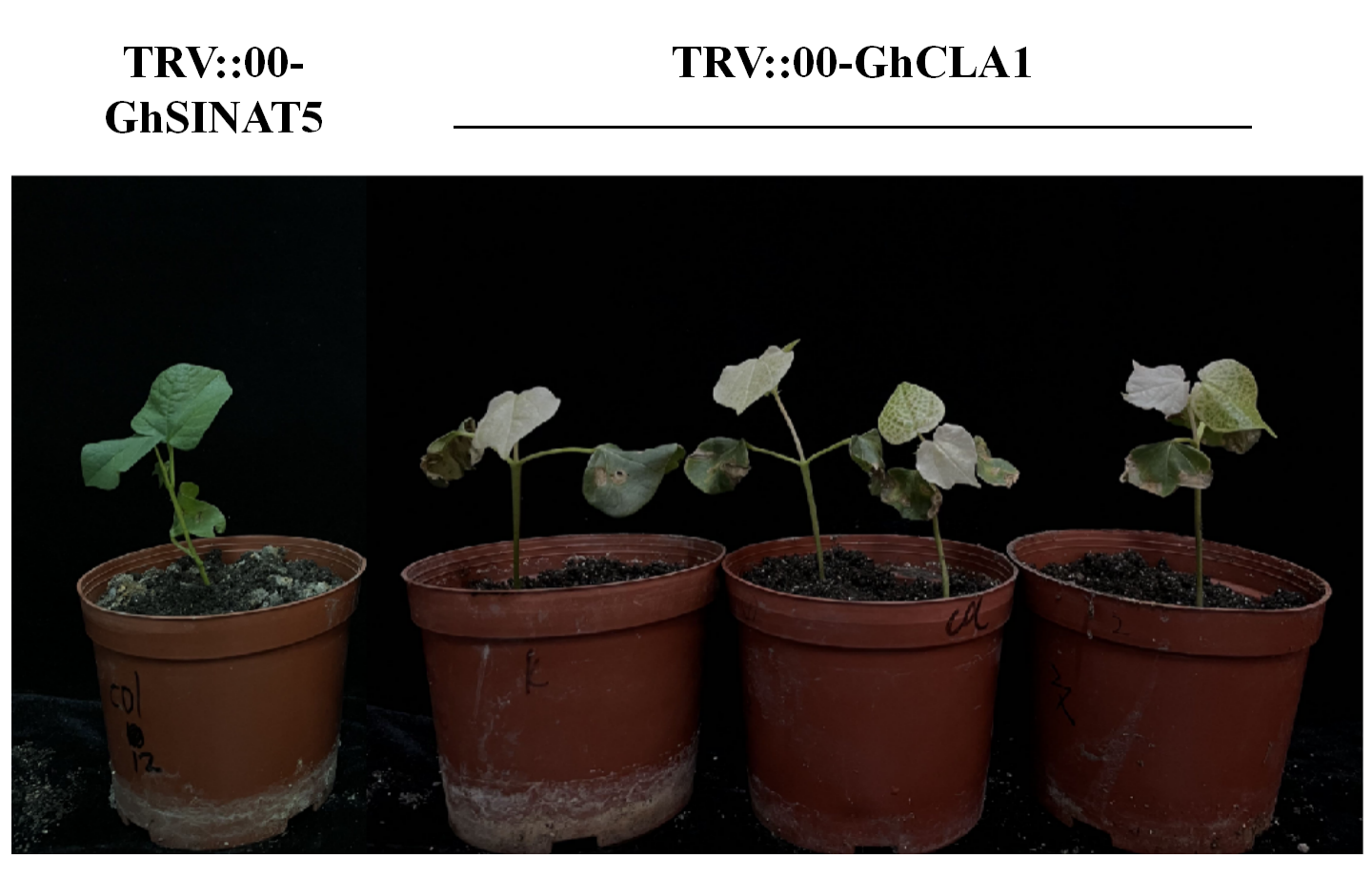

Supplement: Supplementary file 1 [file genes-15-01063-s001.zip › Figures.1.tif]
